# Intermittent fasting combined with calorie restriction is effective for weight loss and cardio-protection in obese women

**DOI:** 10.1186/1475-2891-11-98

**Published:** 2012-11-21

**Authors:** Monica C Klempel, Cynthia M Kroeger, Surabhi Bhutani, John F Trepanowski, Krista A Varady

**Affiliations:** 1 Department of Kinesiology and Nutrition, University of Illinois at Chicago, 1919 West Taylor Street, Room 506F, Chicago, IL, 60612, USA

**Keywords:** Intermittent fasting, Calorie restriction, Liquid diet, Body weight, Visceral fat, Cholesterol, Coronary heart disease, Obese women

## Abstract

**Background:**

Intermittent fasting (IF; severe restriction 1 d/week) facilitates weight loss and improves coronary heart disease (CHD) risk indicators. The degree to which weight loss can be enhanced if IF is combined with calorie restriction (CR) and liquid meals, remains unknown.

**Objective:**

This study examined the effects of IF plus CR (with or without a liquid diet) on body weight, body composition, and CHD risk.

**Methods:**

Obese women (n = 54) were randomized to either the IFCR-liquid (IFCR-L) or IFCR-food based (IFCR-F) diet. The trial had two phases: 1) 2-week weight maintenance period, and 2) 8-week weight loss period.

**Results:**

Body weight decreased more (P = 0.04) in the IFCR-L group (3.9 ± 1.4 kg) versus the IFCR-F group (2.5 ± 0.6 kg). Fat mass decreased similarly (P < 0.0001) in the IFCR-L and IFCR-F groups (2.8 ± 1.2 kg and 1.9 ± 0.7 kg, respectively). Visceral fat was reduced (P < 0.001) by IFCR-L (0.7 ± 0.5 kg) and IFCR-F (0.3 ± 0.5 kg) diets. Reductions in total and LDL cholesterol levels were greater (*P* = 0.04) in the IFCR-L (19 ± 10%; 20 ± 9%, respectively) versus the IFCR-F group (8 ± 3%; 7 ± 4%, respectively). LDL peak particle size increased (P < 0.01), while heart rate, glucose, insulin, and homocysteine decreased (P < 0.05), in the IFCR-L group only.

**Conclusion:**

These findings suggest that IF combined with CR and liquid meals is an effective strategy to help obese women lose weight and lower CHD risk.

## Introduction

Coronary heart disease (CHD) remains the number one of killer of women in the United States [1]. Weight gain during adulthood increases the risk of CHD [1]. Epidemiological evidence suggests that a modest reduction in weight (i.e. 5% of body weight) in female subjects reduces the incidence and progression of CHD [2]. Although several diet strategies exist to help individuals lose weight, one regimen that has gained considerable popularity in the past decade is intermittent fasting (IF) [3]. This diet strategy generally involves severe restriction (75-90% of energy needs) on 1 or 2 days per week. Results from a recent 24-week randomized clinical trial revealed that IF can reduce body weight by 7% in obese women [4]. LDL cholesterol and triglyceride concentrations also decreased by 10% and 17%, respectively, in these subjects [4]. Though these findings are promising, this regimen is limited in that a long duration of time (i.e. 24 weeks) is required to experience only modest reductions in body weight. One possible method of augmenting the rate of weight loss is to combine IF with a daily calorie restriction (CR) protocol. In this way, the individual would fast one day per week, and then undergo mild CR (i.e. 20% restriction of energy needs) on 6 days per week. This combination therapy (IF plus CR) produces greater reductions in weight and superior changes in CHD risk parameters when compared to each intervention alone in animal models [5]. Whether the beneficial effects of IF plus CR on weight and CHD risk can be reproduced in human subjects has yet to be elucidated.

It is well known that many obese subjects are unable to adequately estimate portion sizes [6]. This inability to estimate portions during periods of dieting results in excessive food intake, which then blunts overall weight loss [6]. In order to take the guesswork out of estimating portion sizes, some CR protocols implement portion controlled liquid meals to replace 1 or 2 meals per day. When liquid meal replacements are employed, subjects tend to lose greater amounts of weight when compared to subjects receiving diet counseling alone [7,8]. Whether or not the implementation of liquid meal replacements during periods of IF plus CR accelerates weight loss remains unknown.

Accordingly, the objective of the present study was to examine the effects of an IF protocol combined with CR on body weight, body composition, and CHD risk factors in obese women. Whether the addition of liquid meal replacements to this protocol would result in greater weight loss and more pronounced CHD risk reduction was also assessed.

## Methods

### Subjects

**Figure 1:**
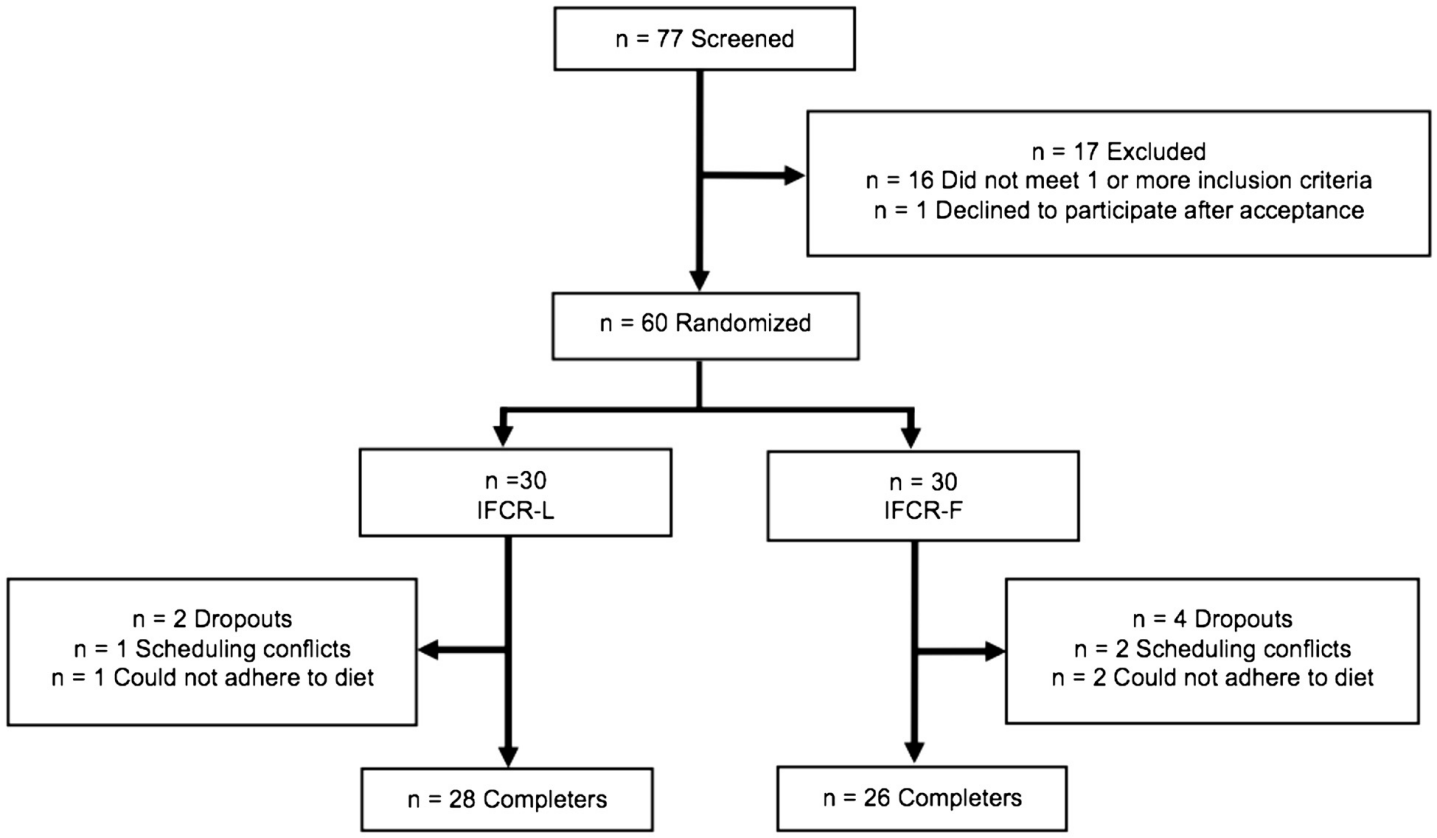
**Study flow chart.** IFCR-L: Intermittent fasting calorie restriction-liquid diet; IFCR-F: Intermittent fasting calorie restriction-food based diet.

Subjects were recruited by means of advertisements placed on and around the University of Illinois campus in downtown Chicago. A total of 77 individuals responded to the advertisements, but only 60 were deemed eligible to participate after the preliminary questionnaire, body mass index (BMI) and waist circumference assessment (Figure 1). Key inclusion criteria were as follows: female, age 35–65 y, BMI between 30 and 39.9 kg/m^2^, waist circumference >88 cm, weight stable for 3 months prior to the beginning of the study (i.e. <5 kg weight loss or gain), non-diabetic, no history of cardiovascular disease, no history of cancer, sedentary or lightly active for 3 months prior to the beginning of the study (i.e. <3 h/week of light-intensity exercise at 2.5–4.0 metabolic equivalents (METS)), non-smoker, not claustrophobic, and not taking weight loss, lipid-lowering, or glucose-lowering medications. Peri-menopausal women were excluded from the study, and post-menopausal women (defined as absence of menses for 2 y) were required to maintain their current hormone replacement therapy regimen for the duration of the study. The experimental protocol was approved by the Office for the Protection of Research Subjects at the University of Illinois, Chicago, and all volunteers gave written informed consent to participate in the trial.

### Experimental design

Randomization was performed by way of a stratified random sample. The sample frame was divided into strata based on BMI and age. Subjects from each stratum were then randomly assigned to either the IFCR-liquid diet (IFCR-L) group (n = 30) or IFCR-food based diet (IFCR-F) group (n = 30). The 10-week trial consisted of two dietary phases: 1) a 2-week baseline weight maintenance period, and 2) an 8-week weight loss period.

### Diet protocol

#### Baseline weight maintenance diet (Week 1–2)

Before commencing the 8-week weight loss intervention, each subject participated in a 2-week baseline weight maintenance period. During this period, subjects were requested to maintain a stable weight and continue eating their usual diet.

#### Weight loss diets (Week 3–10)

Following the baseline period, subjects participated in the IFCR-L or IFCR-F protocol for 8 weeks. Energy requirements were measured using the Mifflin equation [9]. **IFCRL subjects** (n = 30) consumed a calorie-restricted liquid diet for the first 6 days of the week, and then underwent a fast on the last day of the week (water consumption + 120 kcal of juice powder only, for 24 h). The liquid diet (during the CR period) consisted of a liquid meal replacement for breakfast (240 kcal) and a liquid meal replacement for lunch (240 kcal). All liquid meal replacements were provided to the subjects in powder-form in premeasured packets (Isalean Shake, Isagenix LLC, Chandler, AZ). At dinnertime, IFCR-L subjects consumed a 400–600 kcal meal. Food was not provided to the subjects for the dinnertime meal. Instead, subjects met with a Registered Dietician weekly to learn how to make healthy eating choices that are in compliance with the National Cholesterol Education Program Therapeutic Lifestyle Changes (TLC) diet (i.e. <35% of kcal as fat; 50-60% of kcal as carbohydrates; <200 mg/d of dietary cholesterol; and 20–30 g/d of fiber). In following this regimen, each subject was calorie restricted by 30% of their baseline needs. **IFCRF subjects** (n = 30) consumed a calorie-restricted food-based diet for the first 6 days of the week, and then underwent a fast on the last day of the week (water consumption + 120 kcal of juice powder only, for 24 h). IFCR-F subjects ate 3 meals per day in accordance with the TLC diet guidelines. Food was not provided to the subjects. Instead, subjects met with a Registered Dietician weekly to learn how to make healthy eating choices that were in compliance with the TLC diet. Subjects were instructed to eat approximately 240 kcal for breakfast, 240 kcal for lunch, and 400–600 kcal for dinner. In following this regimen, each IFCR-F subject was calorie restricted by 30% of their baseline needs.

## Analyses

### Adherence with diets

A 7-d food record was used to assess adherence to the diets. Subjects completed the food records at week 3 and 10. The Registered Dietician provided 15 min of instruction to each participant on how to complete the food records. These instructions included verbal information and detailed reference guides on how to estimate portion sizes and record food items in sufficient detail to obtain an accurate estimate of dietary intake. All dietary information from the food records was entered into the food analysis program, Nutritionist Pro (Axxya Systems, Stafford, TX) by a single trained operator to alleviate inter-investigator bias. In addition, IFCR-L subjects were provided with a checklist each day to monitor: 1) adherence to the liquid meal protocol, and 2) adherence to the fast day regimen. IFCR-F subjects were also given a checklist to monitor their adherence to the fast day regimen. If an IFCR-L or IFCR-F subject consumed more that 100 kcal on the fast day (above the 120 kcal of juice powder allotted to the subject), the subject was considered non-adherent with the fast day protocol.

### Maintenance of physical activity habits

Changes in daily energy expenditure associated with alterations in physical activity habits were quantified by the use of a pattern recognition monitor (Sense Wear Mini (SWM), Bodymedia, Pittsburgh, PA). Subjects wore the lightweight monitor on their upper arm for 7 d at week 3 and 10 of the trial. The SWM is a wireless multi-sensor activity monitor that integrates motion data from a triaxial accelerometer along with several other physiological sensors (heat flux, skin temperature and galvanic skin response). The data was analyzed using Bodymedia Software V.7.0, algorithm V.2.2.4 (Bodymedia, Pittsburgh, PA).

### Body weight and body composition assessment

Body weight measurements were taken to the nearest 0.5 kg at the beginning of each week in light clothing and without shoes using a balance beam scale (HealthOMeter; Sunbeam Products, Boca Raton, FL). Height was assessed using a wall-mounted stadiometer to the nearest 0.1 cm. BMI was assessed as kg/m^2^. Fat mass and fat free mass were assessed by dual energy X-ray absorptiometry (DXA) at weeks 1, 3 and 10 (QDR 4500 W, Hologic Inc. Arlington, MA). Waist circumference was measured by a flexible tape to the nearest 0.1 cm, midway between the lower costal margin and super iliac crest during a period of expiration. Abdominal visceral adipose tissue and subcutaneous adipose tissue volumes were quantified using magnetic resonance imaging (MRI) at week 3 and 10 [10]. Images were acquired using a 1.5-T superconducting magnet (Siemens, Iselin, NJ), and were obtained every 1 cm from the 9th thoracic vertebra (T9) to the first sacral vertebra (S1). Image locations were defined relative to a common anatomical landmark, the L4–L5 intervertebral space. To facilitate the comparison of individual image data, we limited our analyses to the set of images ranging from 20 cm above L4–L5 (+20 cm) to 3 cm below L4–L5 (−3 cm), for a total of 24 images per subject. Segmentation of the axial images into visceral adipose tissue and subcutaneous adipose tissue areas (cm^2^) was performed using HippoFat image analysis software [11]. Visceral adipose tissue and subcutaneous adipose tissue areas were summed across all 24 images to obtain visceral adipose tissue and subcutaneous adipose tissue volumes, and then these volumes were multiplied by 0.916 g/cm^3^, the density of adipose tissue, to obtain total visceral adipose tissue mass (kg) and total subcutaneous adipose tissue mass (kg) [10].

### Blood collection protocol

Twelve-hour fasting blood samples were collected between 6.00 am and 9.00 am at weeks 1, 3 and 10. The subjects were instructed to avoid exercise, alcohol, and coffee for 24 h before each visit. Blood was centrifuged for 10 min at 520 × g at 4°C to separate plasma from red blood cells and was stored at −80°C until analyzed.

### Plasma lipid and LDL particle size determination

Plasma total cholesterol, direct LDL cholesterol, HDL cholesterol, and triglyceride concentrations were measured in duplicate by enzymatic kits (Biovision Inc, Mountainview, CA). The interassay coefficient of variations (CV) for total cholesterol, LDL cholesterol, HDL cholesterol, and triglycerides were 2.4%, 3.7%, 4.0%, and 3.5%, respectively. LDL particle size was measured by linear polyacrylamide gel electrophoresis (Quantimetrix Lipoprint System, Redondo Beach, CA) [12]. High-resolution 3% polyacrylamide gel tubes were used for electrophoresis. Briefly, 25 μL of sample was mixed with 200 μL of liquid loading gel containing Sudan black, and added to the gel tubes. After photopolymerization at room temperature for 30 min, samples were electrophoresed for 1 h (3 mA/gel tube). Lipoware computer software (Quantimetrix, Redondo Beach, CA) was then used to divide LDL into small (<255 Å), medium (255–260 Å), and large (>260 Å) particles [12].

### Coronary heart disease risk indicator assessment

Blood pressure and heart rate were measured in triplicate each week using a digital automatic blood pressure/heart rate monitor (Omron HEM 705 LP, Kyoto, Japan) with the subject in a seated position after a 10-min rest. Fasting plasma glucose was measured in duplicate at week 1, 3, and 10 with a glucose hexokinase reagent kit (A-gent glucose test, Abbott, South Pasadena, CA; inter-assay CV: 2.8%). Insulin, C-reactive protein (CRP), homocysteine, adiponectin, and leptin were assessed in duplicate at week 1, 3, and 10 by ELISA (R&D Systems, Minneapolis, MN; inter-assay CVs: 3.0%, 3.4%, 3.9%, 4.7%, and 4.2%, respectively).

### Statistics

Results are presented as mean ± SEM. An independent samples t-test was used to test baseline differences between groups. Repeated-measures ANOVA was performed (taking time as the within-subject factor and diet as the between-subject factor) to assess differences between groups over the course of the study. Post-hoc analyses were performed using the Tukey test. Differences were considered significant at P < 0.05. All data was analyzed using SPSS software (version 20.0, SPSS Inc, Chicago, IL).

## Results

### Subject dropout and baseline characteristics

Of the 30 subjects that were randomized to the IFCR-L group, 1 subject dropped out due to scheduling conflicts and 1 subject dropped out because they could not adhere to the diet (Figure 1). Thus 28 subjects completed the IFCR-L protocol. Of the 30 subjects that were randomized to the IFCR-F group, 2 dropped out due to scheduling conflicts and 2 others dropped out because they could not adhere to the diet. Baseline characteristics of the IFCR-L and IFCR-F groups are reported in Table 1. There were no differences between groups for age, ethnicity, menopausal status, waist circumference, BMI, plasma lipids, fasting glucose, or insulin.

**Table 1:** **Subject characteristics at baseline**^**1**^

**Characteristic**	**IFCR**-**L**	**IFCR**-**F**
n	28	26
Age (y)	47 ± 2	48 ± 2
Ethnicity		
African American	16	18
Asian	3	2
Caucasian	4	2
Hispanic	5	4
Pre-menopausal women	16	14
Post-menopausal women	12	12
Body weight (kg)	95 ± 3	94 ± 3
Height (cm)	165 ± 2	164 ± 2
Waist circumference (cm)	103 ± 1	102 ± 3
Body mass index (kg/m^2^)	35 ± 1	35 ± 1
Total cholesterol (mg/dl)	185 ± 8	188 ± 7
LDL cholesterol (mg/dl)	110 ± 7	115 ± 6
HDL cholesterol (mg/dl)	57 ± 4	55 ± 3
Triglycerides (mg/dl)	71 ± 7	81 ± 8
Glucose (mg/dl)	120 ± 2	120 ± 3
Insulin (uIU/ml)	14 ± 1	15 ± 2

^1^ Values reported as mean ± SEM. IFCR-L: Intermittent fasting calorie restriction-liquid diet; IFCR-F: Intermittent fasting calorie restriction-food based diet. No differences between groups for any parameter (Independent samples t-test).

### Adherence to diets and physical activity maintenance

During the weight loss period (weeks 3–10), adherence to the liquid meal protocol was 92 ± 3% in the IFCR-L group over the course of the 8 weeks. IFCR-F subjects achieved their energy restriction goal on 80 ± 2% of the CR days during each week. As for fast day compliance, there were no differences between groups (P = 0.91) (96 ± 4% and 98 ± 3% compliance for the IFCR-F and IFCR-L groups, respectively). The degree of CR achieved during the weight loss period by the IFCR-L group (29 ± 3%) was greater (P < 0.05) than that achieved by the IFCR-F (22 ± 4%). The macronutrient composition of the IFCR-L diet (28 ± 2% kcal from fat, 20 ± 1% kcal from protein, 52 ± 3% kcal from carbs) did not differ from that of the IFCR-F diet (31 ± 2% kcal from fat, 19 ± 2% kcal from protein, 50% ± 3 kcal from carbs) during the 8-week intervention. Alterations in physical activity habits were quantified by the use of a pattern recognition monitor (i.e. an accelerometer). There were no differences in activity energy expenditure between baseline and post-treatment in the IFCR-L group (week 3: 249 ± 28 kcal/d, week 10: 283 ± 27 kcal/d, P = 0.24), and the IFCR-F group (week 3: 246 ± 37 kcal/d, week 10: 258 ± 43 kcal/d, P = 0.63).

### Weight loss and body composition

**Figure 2:**
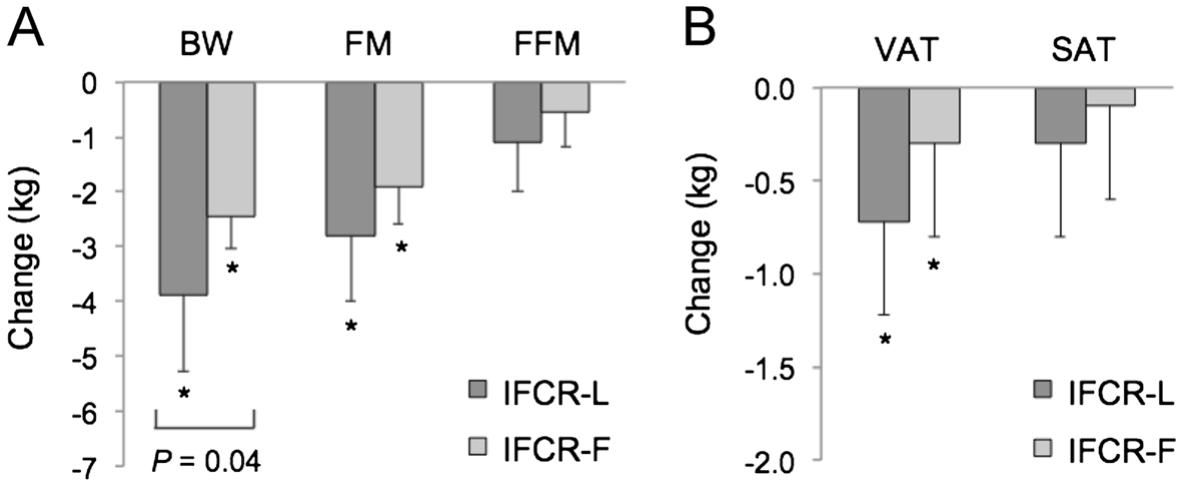
**Body weight and body composition changes during the weight loss period.** IFCR-L: Intermittent fasting calorie restriction-liquid diet (n = 28); IFCR-F: Intermittent fasting calorie restriction-food based diet (n = 26); BW: Body weight; FM: Fat mass; FFM: Fat free mass, VAT: Visceral adipose tissue; SAT: Abdominal subcutaneous adipose tissue. **A**. Changes in body weight, fat mass, and fat free mass in the IFCR-L and IFCR-F groups. IFCR-L group lost more body weight (*P* = 0.04) compared to the IFCR-F group (Repeated-measures ANOVA). *Week 10 absolute values significantly different from baseline (week 3) absolute values, P < 0.0001 (Repeated-measures ANOVA). **B**. Changes in visceral adipose tissue and abdominal subcutaneous adipose tissue in the IFCR-L and IFCR-F groups. *Week 10 absolute values significantly different from baseline (week 3) absolute values, P < 0.001 (Repeated-measures ANOVA).

**Figure 3:**
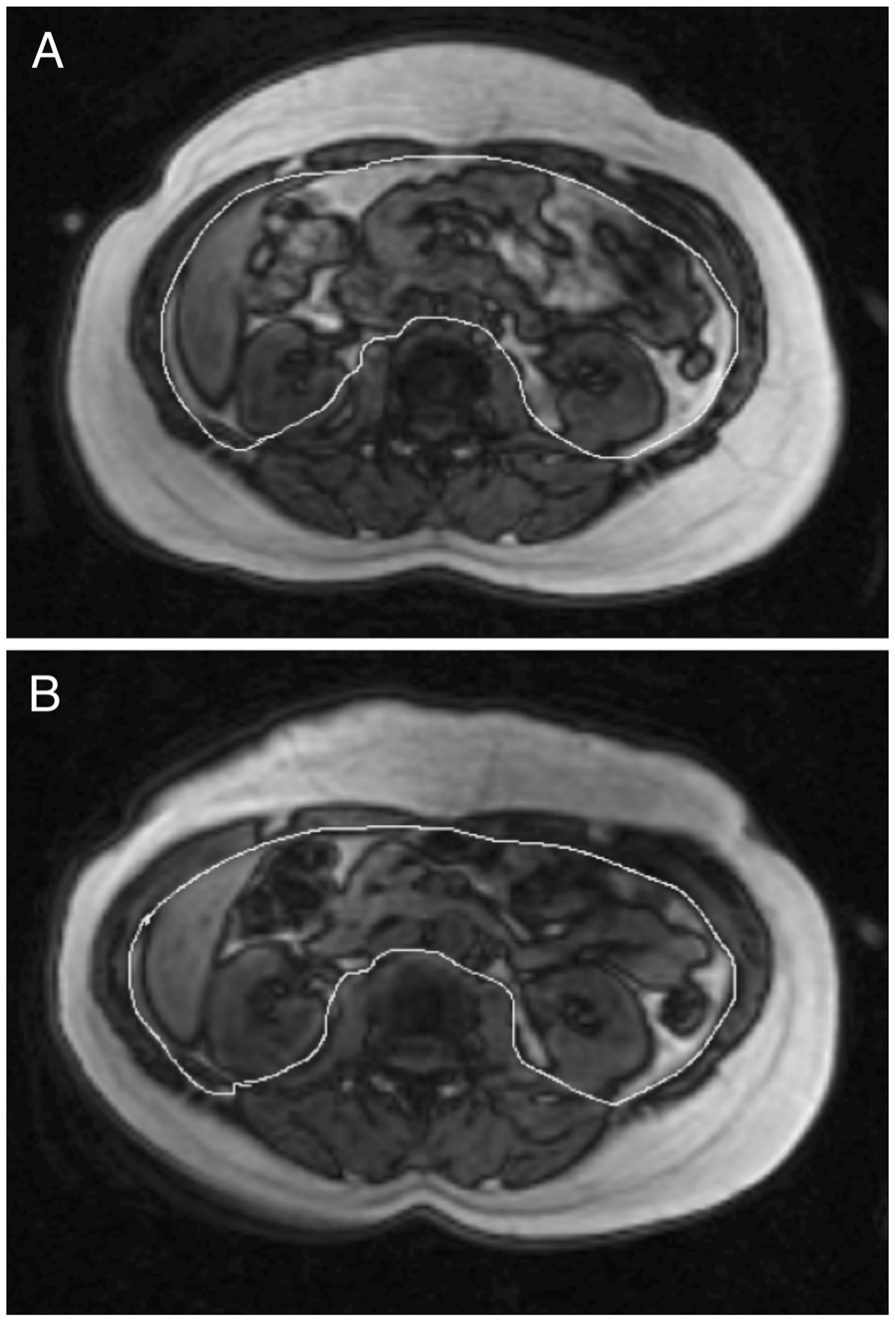
**Change in visceral and subcutaneous adipose tissue area after the IFCR**-**L intervention. A**. Abdominal visceral fat (58 cm^2^) and subcutaneous fat (245 cm^2^) before the intermittent fasting calorie restriction-liquid diet (IFCR-L) at the L3-L4 vertebrae (week 3). **B**. Abdominal visceral fat (17 cm^2^) and subcutaneous fat (173 cm^2^) after the IFCR-L regimen at the L3-L4 vertebrae (week 10).

Changes in body weight and body composition are reported in Figure 2. During the weight maintenance period, body weight remained stable in both the IFCR-L group (week 1: 95 ± 3, week 3: 95 ± 3 kg) and IFCR-F group (week 1: 94 ± 3, week 3: 94 ± 3 kg). During the weight loss period, body weight decreased (P < 0.0001) by 3.9 ± 1.4 kg (4.1 ± 1.5%) in the IFCR-L group and by 2.5 ± 0.6 kg (2.6 ± 0.4%) in the IFCR-F group. Thus, at week 10, body weight of the IFCR-L and IFCR-F group was 91 ± 3 kg and 91 ± 2 kg, respectively. The IFCR-L group lost more body weight compared to the IFCR-F group (P = 0.04). BMI decreased (P < 0.0001) by 1.3 ± 0.5 and 0.8 ± 0.5 kg/m^2^, respectively, in the IFCR-L and IFCR-F groups. Fat mass decreased (P < 0.0001) in the IFCR-L and IFCR-F groups by 2.8 ± 1.2 kg and 1.9 ± 0.7 kg, respectively. Fat free mass remained unchanged throughout the course of the trial in both groups. Visceral fat was reduced (P < 0.001) in the IFCR-L (0.7 ± 0.5 kg) and IFCR-F (0.3 ± 0.5 kg) groups after 8 weeks of treatment. Abdominal subcutaneous fat was not affected by either intervention. There were no differences between groups for fat mass, fat free mass, visceral adipose tissue, or abdominal subcutaneous adipose tissue at any time point. Figure 3 depicts the change in visceral and subcutaneous adipose tissue in one subject in the IFCR-L group before and after the intervention.

### Plasma lipids and LDL particle size

**Figure 4:**
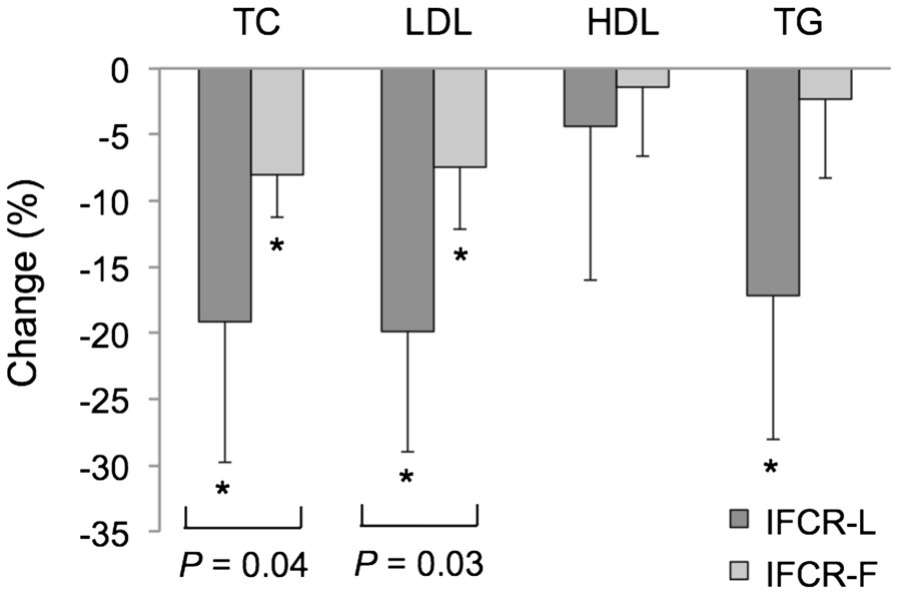
**Plasma lipid changes during the weight loss period.** IFCR-L: Intermittent fasting calorie restriction-liquid diet (n = 28); IFCR-F: Intermittent fasting calorie restriction-food based diet (n = 26); TC: Total cholesterol; LDL: Low density lipoprotein cholesterol; HDL: High density lipoprotein cholesterol; TG: Triglycerides. *Week 10 values significantly different from baseline (week 3) values, P < 0.01 (Repeated-measures ANOVA). IFCR-L group experienced significantly greater reductions in total cholesterol and LDL cholesterol concentrations when compared to the IFCR-F group (*P* < 0.05) (Repeated-measures ANOVA).

Plasma lipids did not change during the baseline period in either the IFCR-L or IFCR-F group. Changes in plasma lipid concentrations during the weight loss period are displayed in Figure 4. Total cholesterol concentrations decreased (P = 0.04) to a greater extent in the IFCR-L group (19 ± 10%) compared to the IFCR-F group (8 ± 3%). LDL cholesterol concentrations were also reduced (P = 0.03) to a greater degree by the IFCR-L diet (20 ± 9%) versus the IFCR-F diet (7 ± 4%). HDL cholesterol was not affected by either intervention. Triglycerides decreased (P < 0.0001) in the IFCR-L group only (17 ± 9%). Changes in LDL particle size characteristics are reported in Table 2. LDL peak particle size increased (P < 0.01) by 2 ± 1 Å in the IFCR-L group only. The proportion of large and medium particles was augmented (P < 0.05) by the IFCR-L diet, but not the IFCR-F diet. The proportion of small particles was reduced (P < 0.05) in the both the IFCR-L (week 3: 37 ± 1%, week 10: 28 ± 2%) and IFCR-F groups (week 3: 39 ± 1%, week 10: 36 ± 1%). However, a greater reduction (P < 0.05) in the proportion of small particles was noted in the IFCR-L group.

**Table 2:** **LDL particle size changes during the weight loss period**^**1**^

		**IFCR**-**L**			**IFCR**-**F**	
	**Week 3**	**Week 10**	**Change**^**2**^	**Week 3**	**Week 10**	**Change**^**2**^
LDL peak size (Å)	260 ± 1	262 ± 1 ^3^	2 ± 1 ^4^	261 ± 1	261 ± 1	0 ± 1
Proportion large particles (%)	34 ± 1	39 ± 1 ^3^	5 ± 2	35 ± 1	37 ± 1	2 ± 1
Proportion medium particles (%)	29 ± 2	33 ± 2 ^3^	4 ± 4	26 ± 2	27 ± 2	1 ± 2
Proportion small particles (%)	37 ± 1	28 ± 2 ^3^	−9 ± 4 ^4^	39 ± 1	36 ± 1 ^3^	−3 ± 1

^1^ Values reported as mean ± SEM. IFCR-L: Intermittent fasting calorie restriction-liquid diet (n = 28); IFCR-F: Intermittent fasting calorie restriction-food based diet (n = 26). Small LDL particles (<255 Å), medium LDL particles (255–260 Å), and large LDL particles (>260 Å).

^2^ Change expressed as the difference between week 3 and week 10 absolute values.

^3^ Week 3 values significantly (P < 0.05) different from week 10 values within group (Repeated-measures ANOVA).

^4^ Absolute change significantly different (P < 0.05) between the IFCR-L and IFCR-F group (Repeated-measures ANOVA).

### Coronary heart disease risk indicators

There were no changes in CHD risk parameters during the weight maintenance period. Changes in CHD risk indices during the weight loss period are displayed in Table 3. Blood pressure was not altered by either the IFCR-L or IFCR-F intervention. Heart rate decreased (P < 0.05) in the IFCR-L group only. Glucose and insulin concentrations decreased (P < 0.05) by the IFCR-L diet, but were not affected by the IFCR-F diet. CRP remained unchanged in both intervention groups. Homocysteine concentrations decreased (P < 0.01) in the IFCR-L group only. Adiponectin and leptin levels were reduced (P < 0.01) by both the IFCR-L and IFCR-F diets.

**Table 3:** **Coronary heart disease risk parameter changes during the weight loss period**^**1**^

		**IFCR**-**L**			**IFCR**-**F**	
	**Week 3**	**Week 10**	**Change**^**2**^	**Week 3**	**Week 10**	**Change**^**2**^
Systolic BP (mm Hg)	120 ± 3	118 ± 2	−2 ± 6	116 ± 4	114 ± 2	−2 ± 4
Diastolic BP (mm Hg)	83 ± 3	79 ± 3	−4 ± 4	80 ± 3	80 ± 2	0 ± 3
Heart rate (bpm)	73 ± 3	70 ± 4 ^3^	−3 ± 4 ^4^	78 ± 2	81 ± 2	3 ± 2
Glucose (mg/dl)	120 ± 2	116 ± 2 ^3^	−4 ± 3	120 ± 3	117 ± 3	−3 ± 4
Insulin (uIU/ml)	14 ± 1	11 ± 1 ^3^	−3 ± 3	15 ± 2	13 ± 2	−2 ± 2
C-Reactive protein (mg/dl)	0.4 ± 0.1	0.4 ± 0.1	0 ± 0.1	0.6 ± 0.2	0.4 ± 0.1	−0.2 ± 0.2
Homocysteine (ng/dl)	10 ± 1	8 ± 1 ^3^	−2 ± 1 ^4^	10 ± 1	10 ± 1	0 ± 1
Adiponectin (ng/ml)	7893 ± 1002	5462 ± 576 ^3^	−2431 ± 802	8442 ± 1201	5931 ± 964 ^3^	−2511 ± 771
Leptin (ng/ml)	37 ± 3	27 ± 3 ^3^	−10 ± 2	38 ± 2	29 ± 2 ^3^	−9 ± 2

^1^ Values reported as mean ± SEM. IFCR-L: Intermittent fasting calorie restriction-liquid diet (n = 28); IFCR-F: Intermittent fasting calorie restriction-food based diet (n = 26); BP: Blood pressure.

^2^ Change expressed as the difference between week 3 and week 10 absolute values.

^3^ Week 3 values significantly (P < 0.05) different from week 10 values within group (Repeated-measures ANOVA).

^4^ Absolute change significantly different (P < 0.05) between the IFCR-L and IFCR-F group (Repeated-measures ANOVA).

## Discussion

Our findings show, for the first time, that the combination of IF plus CR is an effective means of reducing body weight, fat mass, and visceral fat mass in obese women. This novel regimen also decreased key indicators of CHD risk, such as LDL cholesterol, triglycerides, and the proportion of small LDL particles. When liquid meal replacements were incorporated into the IFCR regimen, greater reductions in body weight and indicators of heart disease risk were noted.

Studies of IF in human populations are very limited. To our knowledge, only two studies have examined the effect of IF on body weight [4,13]. In a trial conducted by Williams et al. [13], obese subjects consumed a very-low calorie diet (VLCD; <500 kcal/d) 1 day per week, and ate ad libitum every other day of the week. After 20 weeks of treatment, body weight decreased by 9% (9 kg) from baseline [13]. Similar decreases in body weight were also observed in a recent trial by Harvie et al. [4]. In this study, obese women underwent 2 days of VLCD (600 kcal/d) and ate ad libitum on every other day of the week, for 24 weeks [4]. Body weight was reduced by 7% (6 kg), fat mass decreased by 13% (4 kg), and waist circumference (an indirect indicator of visceral fat) decreased by 6% (6 cm) [4]. In the present study, we observed modest weight loss in both the IFCR-L and IFCR-F groups after 8 weeks of treatment. We also observed that the addition of liquid meals to the protocol resulted in greater weight loss (IFCR-L group: 4.1% weight loss versus IFCR-F group: 2.6% weight loss). Reductions in fat mass and visceral fat mass were also demonstrated at the end of the trial, but did not differ between groups. As for fat free mass, no significant changes were noted in either intervention group. This is surprising as a previous study that implemented energy restricted liquid meals observed small but significant decreases in fat free mass (i.e. 3% reductions from baseline) after 8 weeks of treatment [14]. The greater weight loss by the IFCR-L intervention is most likely due to better dietary adherence. Analysis of food intake revealed a greater degree of energy restriction in the IFCR-L group (29%) compared to the IFCR-F group (22%) over the course of the trial. This greater overall restriction in the IFCR-L group, and hence better adherence, is not surprising as these subjects were given portion-controlled liquid meals for breakfast and lunch everyday. Providing such meals has been shown to boost initial weight loss during dietary restriction protocols as it takes the guesswork out of having to estimate calories from varying foods [7]. Although these liquid meals are effective for helping with initial weight loss, lasting weight loss and weight maintenance requires extensive dietary counseling to instill healthy behaviors that can be employed long-term [8,15]. In view of this, both groups met with a Registered Dietician weekly to incorporate TLC dietary guidelines into their daily lives and to help make the leap from the liquid diet to a wholesome food-based regimen after the study was over.

Beneficial modulations in key lipid risk factors were also observed by both diets. For instance, total and LDL cholesterol decreased in the IFCR-L group (19% and 20%, respectively) and IFCR-F group (8% and 7%, respectively), with greater changes noted for IFCR-L. In contrast, only the IFCR-L group experienced reductions in triglycerides (17% from baseline). The proportion of small LDL particles was also decreased by both the IFCR-L group (week 3: 37 ± 1%, week 10: 28 ± 2%) and IFCR-F group (week 3: 39 ± 1%, week 10: 36 ± 1%). However, increases in LDL peak particle size and the proportion of large LDL particles, were only noted in the IFCR-L group. Taken together, these results suggest that IF combined with CR is an effective means of improving lipid profile in a short-term (8 week) intervention. We also show that adding a liquid diet component may enhance this lipid-lowering effect. The greater decreases in plasma lipids by the IFCR-L diet is most likely due to the greater weight loss noted in this group. For every kg of body weight loss, LDL cholesterol is estimated to decrease by 2 mg/dl [16]. Since the IFCR-L group lost 1.4 kg more body weight than the IFCR-F group, this may explain why the reductions in LDL cholesterol by the liquid diet intervention were more pronounced. The decreases in lipids demonstrated in the present study are similar to what has been reported in previous trials of IF [4,13]. Williams et al. [13] noted a 10% and 52% lowering of LDL cholesterol and triglycerides, respectively, after 20 weeks of treatment. In accordance with these findings, Harvie et al. [4] observed a 10% decrease in LDL cholesterol and a 17% reduction in triglycerides. No trial to date has examined the effect of IF on LDL particle size, thus there is no data for which to compare our findings.

Modulations in other CHD risk parameters were also more pronounced in the IFCR-L group compared to the IFCR-F group. For instance, fasting plasma glucose and insulin were only decreased by the liquid intervention, suggesting that this diet therapy may benefit glycemic control. Heart rate and homocysteine concentrations were reduced solely in the IFCR-L group. Leptin concentrations, on the other hand, were lowered by both diets. The decreases in leptin are most likely mediated by the reductions in fat mass and visceral fat mass observed in both groups [17]. Leptin may be involved in atherosclerotic plaque formation through its effect on cholesterol biosynthesis in monocytes [18]. Thus, these reductions in leptin by IFCR may play a systemic anti-atherogenic role [19]. Adiponectin levels were also decreased by both diet interventions. This finding is not surprising, as adiponectin levels have been shown to decrease during the first 8–12 weeks of CR, and then increase only once a 10% weight loss has been achieved [20]. Since the present trial only ran for 8 weeks, and since weight loss was <5%, this may explain why adiponectin concentrations were lowered from baseline. Blood pressure and CRP also remained unchanged throughout the course of the trial in both groups. Accumulating evidence suggest that a 5 and 10% reduction in body weight is required to decrease blood pressure and CRP, respectively [21,22]. This degree of weight loss was not attained, which may explain why these two variables were unaffected by either treatment.

This study is limited in that it did not tease apart the effect of IF and CR on body weight and CHD risk. Thus, the independent contributions of the IF diet versus the CR diet on these various parameters, are not known. In order to answer these key questions, a future study should be performed that compares the effect of IF combined with CR, to that of IF alone, and CR alone. An additional limitation of the study was that it did not carefully control for food intake by providing food-based meals to the intervention groups (i.e. dinner meal for the IFCR-L subjects, and 3 meals/d for the IFCR-F subjects). If meals were provided, a more precise measurement of energy restriction and dietary adherence could have been obtained. The study is also limited in that it employed HippoFat software to quantify visceral fat mass from MRI images. This software is limited in that it underestimates visceral fat and overestimates subcutaneous fat, particularly in larger individuals [11]. Another limitation of the study is that we employed food records to estimate overall calorie restriction in each group. It is well known that obese individuals underreport food intake by 20-40% when completing food records [23]. Future studies in this field should therefore implement more robust measures of energy assessment such as the doubly-labeled water technique [24]. The last disadvantage of the study is that only female subjects were employed, and as such, the applicability of these findings to males remains uncertain.

In summary, these findings suggest that IFCR may be effective for reducing body weight, visceral fat mass, and CHD risk in obese women. We also report that incorporating liquid meal replacements into an IFCR regimen may facilitate greater weight loss and lipid-lowering. From a clinical standpoint, we would recommend this diet to individuals who wish to boost the weight loss they see with IF, by adding a daily CR regimen. This combination may also help reduce the boredom typically associated with attempting only one dietary plan. Although these short-term findings are promising, the long-term effects of this novel diet strategy still require confirmation in a large-scale human trial.

## Competing interests

Krista Varady has a consulting relationship with the sponsor of the research, lsagenix, LLC.

## Authors’ contributions

MCK conducted the clinical trial, analyzed the data, and assisted with the preparation of the manuscript. CMK assisted with the conduction of the clinical trial and performed the laboratory analyses. SB and JFT performed the laboratory analyses, and assisted with data analyses. KAV designed the experiment and wrote the manuscript. All authors read and approved the final manuscript.

## Funding source

This study was funded by Isagenix LLC., Chandler, AZ
